# Insulin sensitivity and cardiovascular risk after revascularization: associations with remodeling and long-term outcomes

**DOI:** 10.3389/fcvm.2026.1796332

**Published:** 2026-07-27

**Authors:** Ruixuan Tang, Rui Xi, Xiao Zong, Roubai Pan, Suyi Jia, Yan Xu, Rong Tao, Qin Fan

**Affiliations:** 1 Department of Cardiology, Ruijin Hospital, Shanghai Jiao Tong University School of Medicine, Shanghai, China; 2 Institute of Cardiovascular Diseases, Shanghai Jiaotong University School of Medicine, Shanghai, China

**Keywords:** cardiovascular mortality (CVM), insulin sensitivity, ischemic cardiomyopathy, QUICKI, ventricular remodeling

## Abstract

**Introduction:**

Insulin resistance may influence myocardial recovery after ischemic injury, but its relevance to post-revascularization remodeling and long-term outcomes remains unclear.

**Methods:**

We examined 732 patients with ischemic cardiomyopathy who underwent revascularization and had complete metabolic and echocardiographic data, with a median follow-up of 8.1 years. Insulin sensitivity was assessed using the Quantitative Insulin Sensitivity Check Index (QUICKI). Ventricular remodeling was evaluated by changes in LVEDD and LVESD.

**Results:**

Across penalized regression and complementary machine-learning analyses, QUICKI consistently showed a stronger association with cardiovascular mortality than fasting glucose, insulin, or lipid measures. Lower QUICKI values were linked to greater ventricular dilatation, and these associations remained stable across analytic frameworks. Time-dependent ROC analysis demonstrated that QUICKI provided high early discriminatory performance for cardiovascular death. External validation in NHANES 2013-2016 further supported an inverse relationship between QUICKI and cardiovascular mortality risk.

**Discussion:**

These findings suggest that insulin sensitivity, captured by a simple fasting-based index, reflects a metabolic vulnerability that relates to both structural remodeling and long-term prognosis after revascularization, aligning with the metabolic dimension of the CKM framework and offering a potential addition to contemporary risk-stratification strategies.

## Graphical Abstract

**Figure:**
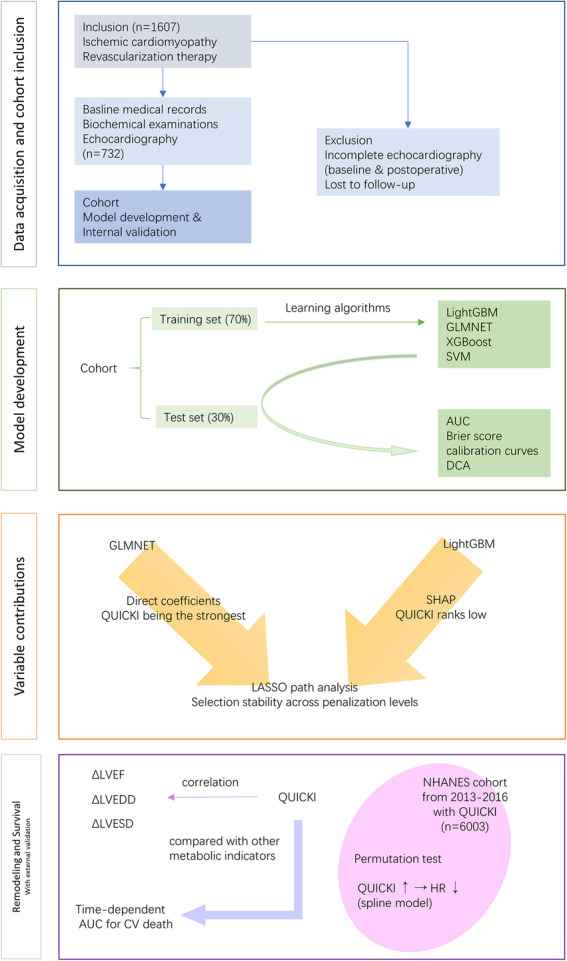
This figure summarizes the analytic workflow and key findings of the study. A cohort of 732 patients with ischemic cardiomyopathy undergoing revascularization was used for model development and internal validation. Multiple machine learning models were trained and evaluated using standard metrics. Variable importance was assessed via GLMNET coefficients, SHAP values, and LASSO path stability. QUICKI emerged as a robust metabolic predictor, outperforming conventional indicators in early cardiovascular mortality prediction and showing consistent associations with ventricular remodeling. External validation in NHANES 2013–2016 confirmed its prognostic relevance.

## Introduction

Cardiac remodeling after ischemic injury is a central determinant of long-term outcomes in ischemic cardiomyopathy (1). Yet remodeling represents a heterogeneous biological process (2), and its prognostic interpretation can be difficult when relying solely on structural measurements (3). Metabolic dysregulation, particularly insulin resistance, may influence myocardial recovery and ventricular adaptation (4), but its contribution to remodeling in revascularized ischemic cardiomyopathy remains insufficiently characterized.

Among the available measures of insulin sensitivity, the Quantitative Insulin Sensitivity Check Index (QUICKI) provides a particularly suitable option for this study. QUICKI has been rigorously validated against the hyperinsulinemic–euglycemic clamp and shows strong linearity and high predictive accuracy across non-obese, obese, hypertensive, and type 2 diabetic populations (5, 6). In calibration analyses involving 116 clamp studies, QUICKI demonstrated the lowest prediction error and the narrowest residual distribution, outperforming HOMA-IR, 1/HOMA, fasting insulin, and even minimal-model indices derived from frequently sampled intravenous glucose tolerance tests (5). Meanwhile, OGTT-based measures such as the Matsuda index require full oral glucose tolerance testing, which is rarely performed in patients with ischemic cardiomyopathy and is generally unavailable in cardiac cohorts; moreover, glucose-insulin ratios during OGTT have shown limited correlation with clamp-measured insulin sensitivity (7). The triglyceride-glucose (TyG) index, while practical when insulin is not measured, reflects a lipid-glucose metabolic axis rather than insulin-mediated myocardial energy metabolism and therefore captures a biologically distinct pathway (8). Therefore, QUICKI provides a simple measure of insulin sensitivity and has been linked to cardiometabolic risk across diverse populations (6, 9, 10). However, whether QUICKI offers prognostic information that is independent of established clinical, metabolic, and structural markers remains unknown. Evaluating this requires analytic frameworks capable of objectively identifying key predictors from correlated domains and determining whether QUICKI contributes unique information beyond conventional risk factors.

Modern machine-learning models were applied not primarily for prediction but for contextual interpretation, offering an alternative perspective on variable importance and enabling comparison across different analytic frameworks (11). These flexible approaches emphasize complex, nonlinear interactions and highlight subgroup-specific pathways (12). To complement this, penalized regression, particularly elastic-net regularization, provided a principled method for identifying stable, independent associations (13). The LASSO coefficient path further enabled evaluation of variable entry, persistence, and selection stability across penalty levels, thereby clarifying whether QUICKI contributes independently to global linear effects (14).

In this study, we integrated metabolic markers, echocardiographic remodeling measures, and both penalized regression and machine-learning interpretive analyses to clarify how insulin sensitivity relates to ventricular remodeling and long-term outcomes after revascularization. We compared QUICKI against conventional metabolic indicators to assess its relative prognostic strength, and further validated its associations in an independent population-based cohort [National Health and Nutrition Examination Survey (NHANES) 2013–2016]. This multimethod framework was designed to explore the interplay between metabolic dysfunction, ventricular remodeling, and prognosis in ischemic cardiomyopathy, providing deeper mechanistic insights into disease progression.

## Materials and methods

### Study population and cohort definition

We derived a retrospective cohort from inpatient and follow-up databases at Shanghai Ruijin Hospital (2010–2018). This cohort comprised 732 patients with ischemic cardiomyopathy who underwent revascularization [percutaneous coronary intervention (PCI), coronary artery bypass grafting (CABG), or hybrid procedures] and had complete baseline records, long-term follow-up (median 8.1 years), and postoperative echocardiography performed within 3 years after surgery; Only patients with available postoperative echocardiography within 3 years after revascularization were eligible (Figure Graphical Abstract). The final sample size reflected patients in our institutional cohort who had complete clinical, laboratory, and imaging data available during the study period. This strictly defined subset was used for model development and internal validation because of its data completeness. The primary endpoint was cardiovascular death, ascertained through outpatient records or telephone interviews.

### Ethics and data collection

The study protocol was approved by the institutional ethics committee of Shanghai Ruijin Hospital (number 2016–019). All medical records and follow-up data were retrieved by trained personnel from the hospital's electronic medical record system. To reduce bias, data collection and statistical analysis were conducted independently. All data were anonymized prior to analysis and processed under hospital privacy and data-sharing regulations. This study was conducted in accordance with the STROBE (Strengthening the Reporting of Observational Studies in Epidemiology) statement. The completed STROBE checklist is provided in the Supplementary Material.

### Baseline variables and metabolic indices

Baseline variables included key clinical, laboratory, and echocardiographic measures. To provide a more comprehensive metabolic characterization, both traditional and derived indices were incorporated. Traditional indicators comprised fasting glucose, fasting insulin, triglycerides (TG), total cholesterol (TC), high-density lipoprotein cholesterol (HDL-C), low-density lipoprotein cholesterol (LDL-C), and body mass index (BMI). Insulin sensitivity was further estimated using QUICKI, calculated from fasting glucose (mmol/L) and insulin (*μ*U/mL) concentrations, as follows (6):

$$\begin{array}{rl} QUICKI\;=\\ & \;\;\frac{1}{\log(\;fasting\;insulin[\mu U/mL])+\;\log(\;fasting\;glucose[mmol/L])} \end{array}$$

In addition, other derived indices reflecting systemic inflammation and related biological processes were also included.

### Data preprocessing and imputation

Random seeds were set to ensure reproducibility. Samples were randomly divided into training and test sets (70:30). Missingness patterns did not indicate systematic missing, supporting the use of simple imputation, and missing values for continuous variables were imputed using the median and for categorical variables using the mode within the training set (15); the imputation rules were also applied to the test set.

### Machine learning models and fusion

We trained four machine learning models: light gradient boosting machine (LightGBM/LGB) (16), penalized generalized linear model (GLMNET) (17), extreme gradient boosting (XGBoost/XGB) (18), and support vector machine (SVM) (19). For model fusion, we emphasized the relative contributions of individual base learners by applying *post-hoc* weighting schemes, primarily using grid-search-based optimization of model weights (20). These exploratory analyses were used for descriptive purposes. Model performance was further assessed using area under the curve (AUC), the Brier score, calibration curves, and decision curve analysis (DCA).

### Regression and feature importance analyses

Regression coefficients were extracted from the GLMNET regularized linear model to quantify direct contributions. LASSO path analysis was performed to evaluate selection stability across varying penalization levels. Cross-validation recorded variable selection under *λ*.min (minimizing mean deviance) and *λ*.1se (simplest model within one standard error of the minimum). SHapley Additive exPlanations (SHAP) values were derived from the fitted LightGBM model using the full contribution matrix, to characterize global feature importance.

### Time-dependent ROC and remodeling associations

Time-dependent Receiver Operating Characteristic (ROC) analyses were used to evaluate the prognostic performance of metabolic indicators, with AUC values calculated at prespecified time points (4–10 years). Associations with cardiac remodeling were examined using linear regression models, reporting regression coefficients, standard errors, and *p*-values.

### External validation in NHANES

For external validation, NHANES 2013–2016 participants with available data to derive QUICKI were included (*n* = 6,003). Cardiovascular death was the endpoint. Between-group differences in QUICKI were tested using the Fisher-Pitman permutation test (10,000 resamples). Restricted cubic spline Cox regression was applied to model the association between QUICKI and cardiovascular mortality, estimating hazard ratios (HRs) with 95% confidence intervals across the distribution.

### Statistical software

All statistical analyses were performed in the R environment (version 4.4.2). Key packages included caret, xgboost, lightgbm, glmnet, and pROC. The study design and analytic workflow are presented in Figure Graphical Abstract.

## Results

### Baseline characteristics

A total of 732 patients with ischemic cardiomyopathy were enrolled, among whom 128 events were recorded during follow-up. With comparable sex distribution between groups, patients in the death group were older and showed worse cardiac and renal function, with elevated blood urea nitrogen (BUN)/creatinine, increased N-terminal pro-B-type natriuretic peptide (NT-proBNP), reduced estimated glomerular filtration rate (eGFR), and enlarged left ventricular end-diastolic diameter (LVEDD), left ventricular end-systolic diameter (LVESD), and left atrial diameter. Both baseline and follow-up left ventricular ejection fraction (LVEF) were lower, and coronary left main disease was more common, while family history showed no difference (Supplementary Table 1). Baseline analyses highlighted significant differences in cardiac remodeling and renal indices between survival and death groups, whereas routine metabolic markers (fasting glucose, insulin, lipids) did not differ. These findings suggest that structural and renal indices reflect overt disease burden, while standard metabolic markers may miss subtler risks. Given the known role of metabolic dysregulation in ischemic cardiomyopathy, machine learning was applied to assess the prognostic value of metabolic variables within the broader context of remodeling and systemic risk.

### Predictive modeling performance and complementarity of linear and tree-based models

**Figure 1:**
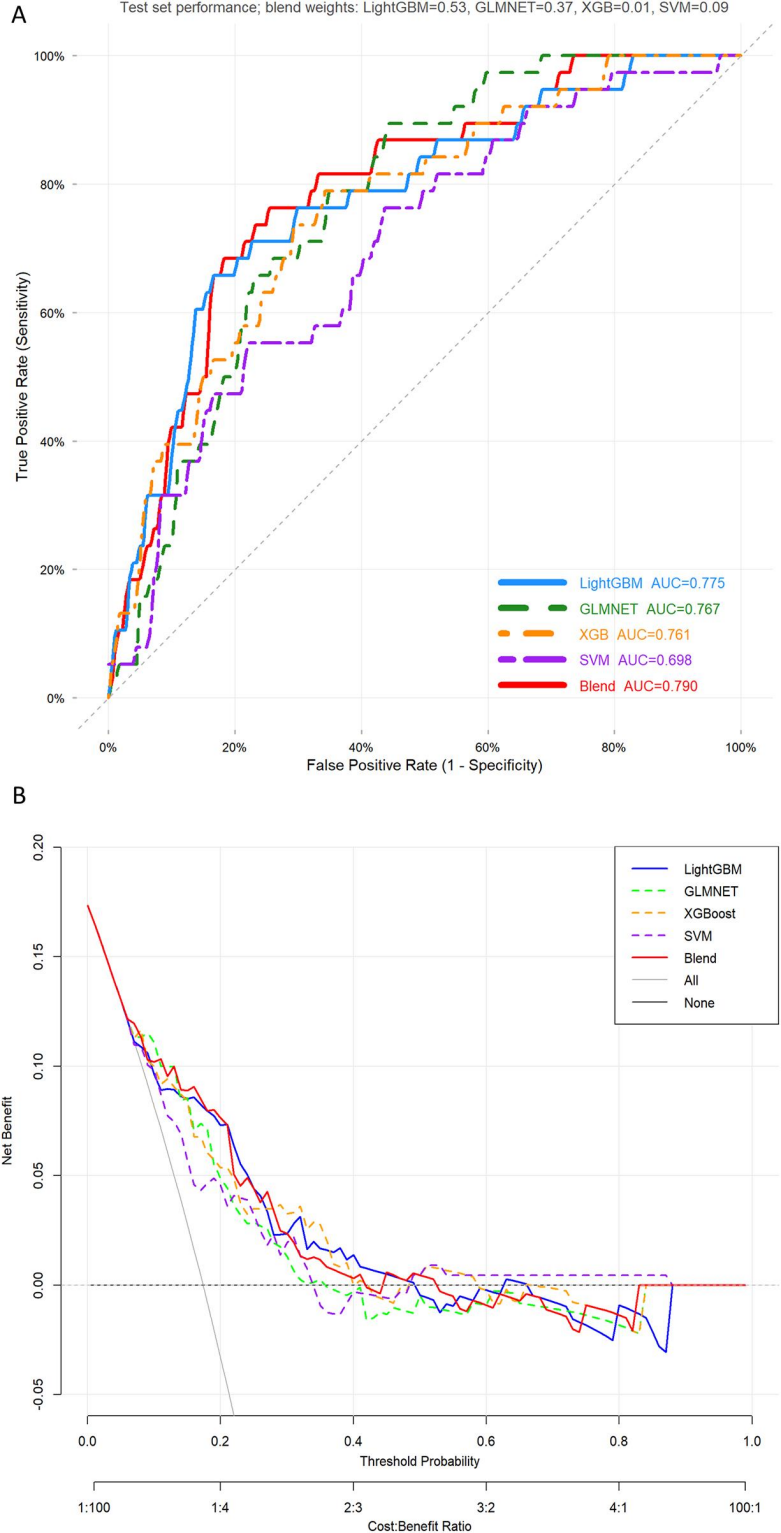
Predictive modeling performance across individual models and their ensemble. **(A)** AUC for each model and the weighted ensemble (Blend), demonstrating comparative discrimination. **(B)** DCA illustrating net clinical benefit across threshold probabilities.

We constructed and compared multiple machine-learning models to predict adverse events and to integrate diverse clinical domains into a unified risk-stratification framework. After splitting the sample into training and testing sets (70/30), multiple single models and their weighted ensemble (Blend) were evaluated on the test set. Grid-search weights were used to visualize relative model contributions, the blending weights determined through grid search assigned 53% to LightGBM, 37% to GLMNET, 1% to XGBoost, and 9% to SVM, with LightGBM and GLMNET contributing the largest proportions. This reflects their complementary strengths (Supplementary Table 2): LightGBM provided the highest sensitivity and favorable negative predictive value, making it suitable for clinical alerts and recall of high-risk patients; GLMNET achieved superior specificity and positive predictive value, making it more appropriate for reducing false positives and confirmatory decision-making. XGBoost and SVM contributed minimally. In summary, LightGBM and GLMNET each offer distinct advantages in different clinical scenarios, and their complementarity is the key driver of the ensemble's enhanced performance. These findings were consistently supported by comparative performance tables, ROC curves, DCA, and calibration plots (Supplementary Table 2, Figure 1, Supplementary Figure 1).

### Variable evaluation: QUICKI as a strong variable

**Figure 2:**
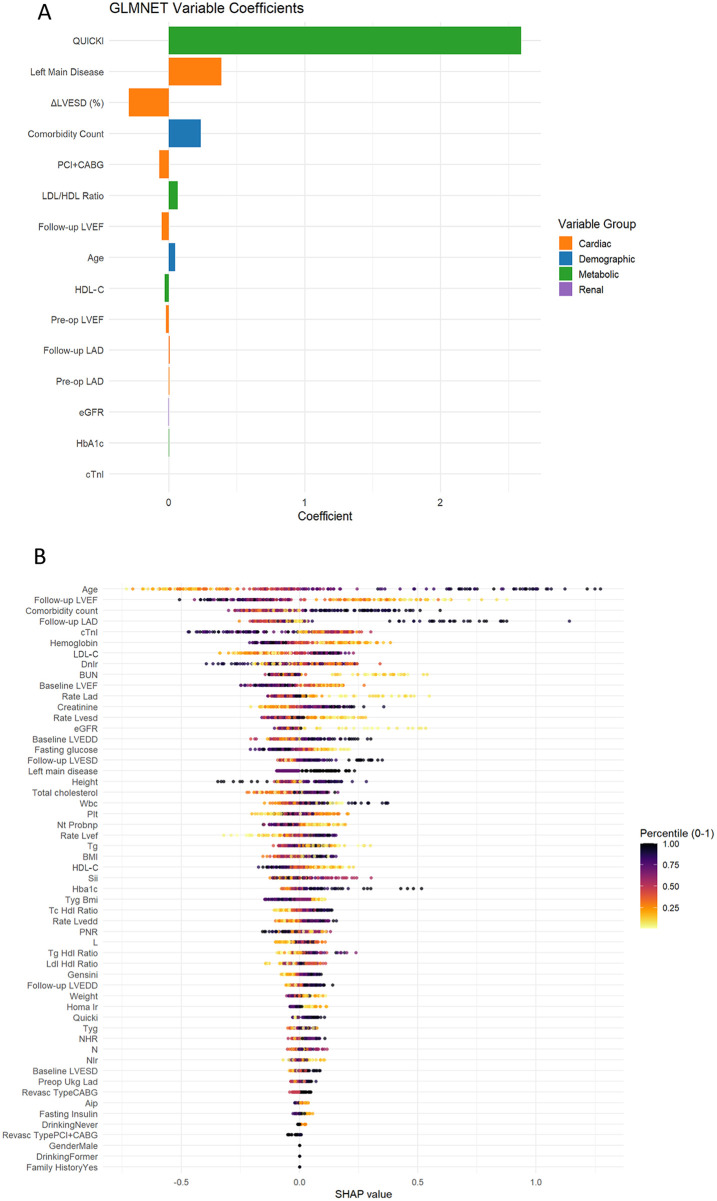
Variable evaluation. **(A)** GLMNET regression coefficients, with variables color-coded by domain (cardiac, demographic, metabolic, and renal). **(B)** SHAP analysis of LightGBM. Features are ordered top to bottom by mean |SHAP|. Each point is a sample; color shows feature percentile across samples.

To assess the prognostic relevance of candidate variables, we first examined their immediate contributions through regression coefficients and then evaluated their stability along the LASSO selection path, thereby determining whether predictors such as QUICKI consistently entered the model across varying penalization levels. QUICKI emerged as the most prominent predictor, with the largest positive coefficient (*β* = 2.59), highlighting its strong association with survival outcomes (Figure 2A). Other variables with moderate coefficients included left main disease, LVESD change rate, and comorbidity count, while age, follow-up LVEF showed smaller effects. We then applied SHAP analysis to LightGBM to capture nonlinear feature importance (Figure 2B). In contrast to GLMNET, LightGBM emphasized age, follow-up LVEF, left atrial diameter, creatinine, and cardiac troponin I (cTnI) as the most influential predictors, whereas QUICKI ranked considerably lower. This divergence indicates that the linear framework highlights the independent contribution of metabolic indices such as QUICKI and tends to select variables with higher generalizability, whereas the tree-based model relies more heavily on complex interactions among cardiac remodeling, renal function, and myocardial injury markers.

### Stability of QUICKI in penalized regression

**Figure 3:**
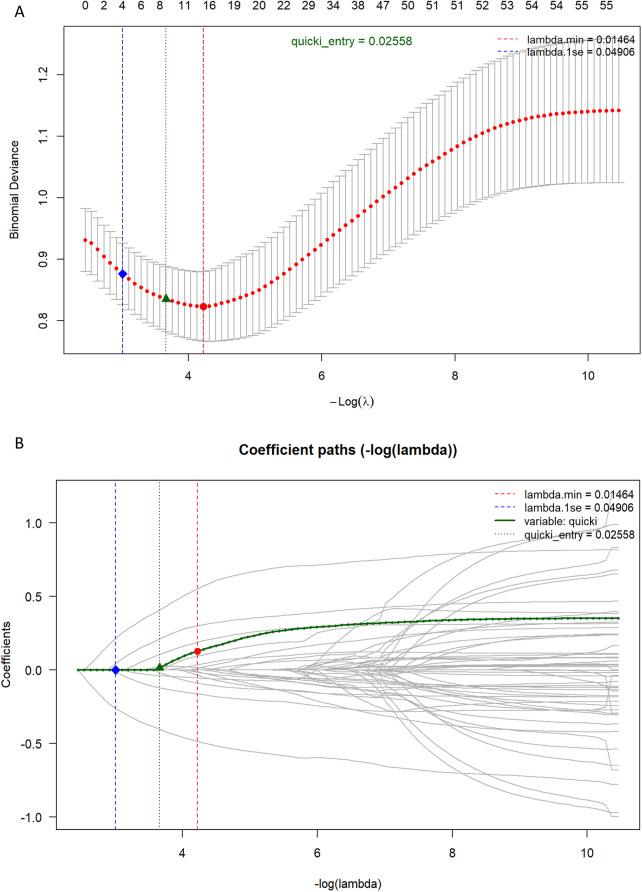
Stability of variable selection in penalized regression. **(A)** Five-fold cross-validation curve for LASSO within GLMNET. **(B)** LASSO regression path, illustrating variable entry and coefficient trajectories across penalty levels.

Then we conducted an in-depth analysis of the LASSO regression path and cross-validation results within GLMNET (Figure 3). Core clinical variables showed the highest and most stable selection frequencies across penalty levels, while structural parameters fluctuated along the *λ* path but still added explanatory value (Supplementary Table 3). Within this framework, the performance of QUICKI was noteworthy: its entry point was located between *λ*.min and *λ*.1se (entry ≈ 0.0256), suggesting that it could be incorporated into the model within a reasonable penalty range. At *λ*.min, QUICKI reached an entry frequency of 0.73, demonstrating stable inclusion under optimal penalization (Supplementary Table 3). Although its overall entry frequency was slightly lower than that of core clinical variables, QUICKI consistently appeared in the model as a representative metabolic index, highlighting its stable selection within GLMNET and underscoring its contribution to mortality risk prediction.

### QUICKI outperforms conventional metabolic indicators in early prognosis

**Figure 4:**
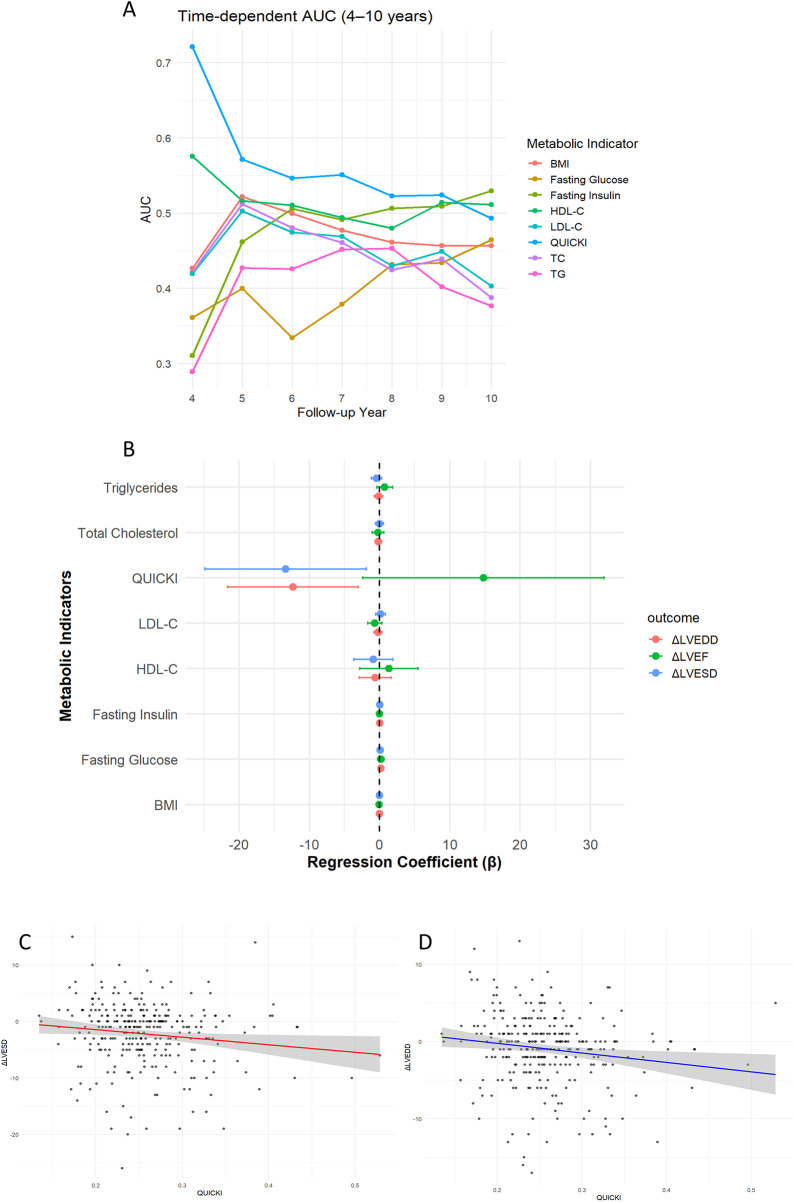
Prognostic performance and association of QUICKI with cardiac remodeling. **(A)** Time-dependent AUC curves for cardiovascular death over 4-10 years, comparing QUICKI with conventional metabolic indicators. **(B)** Forest plot of linear coefficients and 95% confidence intervals for metabolic variables in relation to cardiac remodeling. **(C)** Scatter plot of QUICKI vs. *Δ*LVESD. **(D)** Scatter plot of QUICKI vs. *Δ*LVEDD.

Building on the machine-learning findings that highlighted QUICKI as an important metabolic marker, we next examined its prognostic value in relation to cardiovascular death. In time-dependent ROC analysis, QUICKI demonstrated the strongest discriminatory ability for cardiovascular death at 4 years of follow-up (Figure 4A, Supplementary Table 4). Compared with conventional metabolic indicators, QUICKI consistently showed superior or comparable prognostic performance across subsequent time points. Although its predictive ability declined over longer follow-up, the early advantage of QUICKI highlights its potential as a more sensitive marker of adverse cardiovascular outcomes than traditional metabolic measures such as fasting glucose, insulin, lipids, or BMI.

### Association of QUICKI with cardiac remodeling

We further examined its relationship with cardiac remodeling. QUICKI showed clear inverse associations with changes in LVEDD and LVESD (Supplementary Table 5), indicating that reduced insulin sensitivity was linked to progressive ventricular dilatation. Visual analyses reinforced these findings: scatter plots demonstrated consistent inverse relationships between QUICKI and ventricular dimensions (Figures 4C,D), and forest plots highlighted QUICKI as the only metabolic indicator with robust associations (Figure 4B). In contrast, conventional measures showed limited predictive value.

### External validation of QUICKI in NHANES

**Figure 5:**
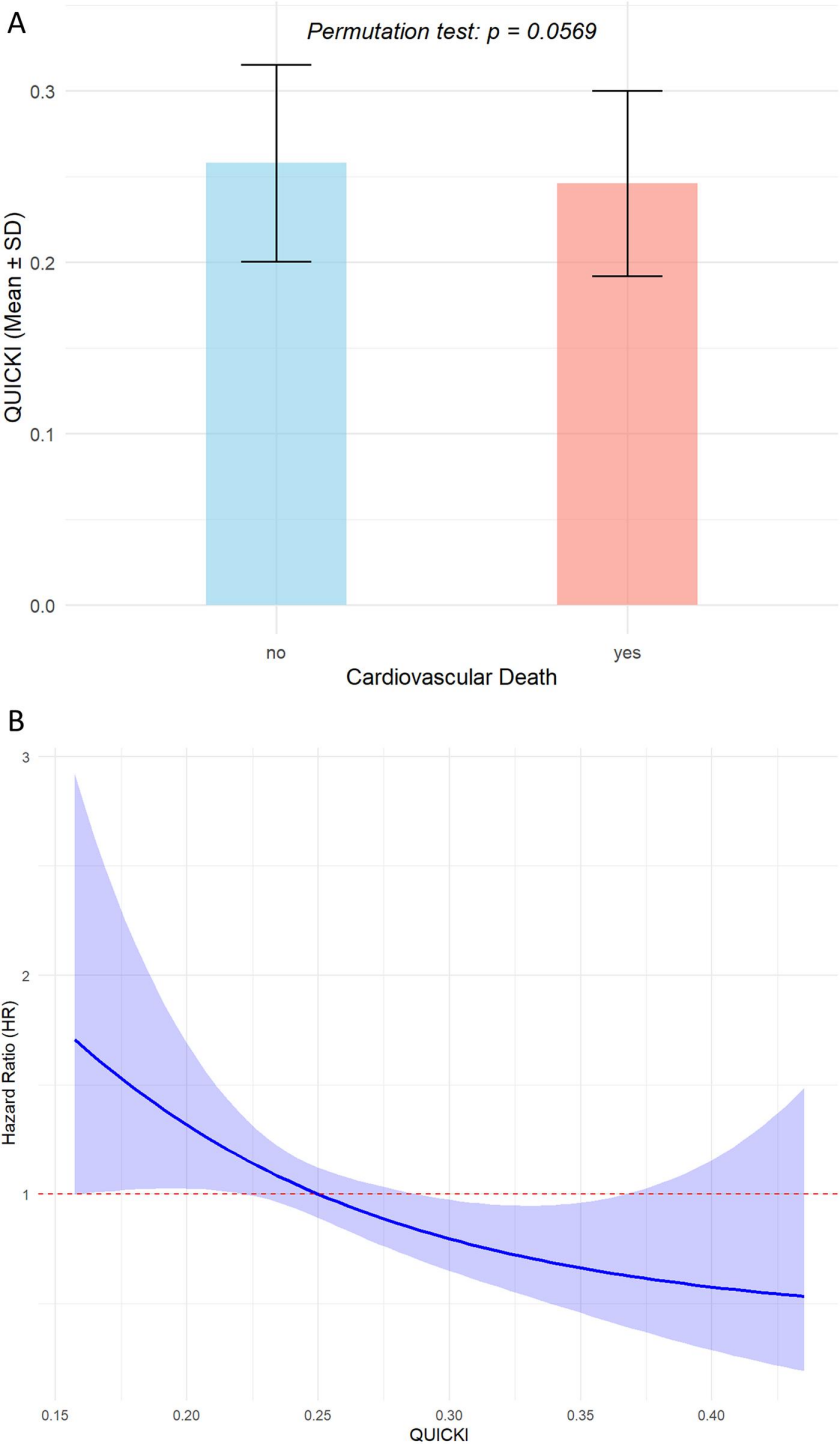
External validation of QUICKI. **(A)** Boxplot of baseline QUICKI by cardiovascular death status. **(B)** Spline-based Cox regression of QUICKI and cardiovascular mortality risk.

Finally, to assess generalizability, we performed external validation in NHANES 2013–2016. And 6,003 participants with sufficient data to calculate QUICKI were included. Baseline characteristics showed that the mean QUICKI was 0.258 ± 0.057 in those without cardiovascular death (*n* = 5,920) and 0.246 ± 0.054 in those with cardiovascular death (*n* = 83). The Fisher-Pitman permutation test indicated a borderline difference between groups (*p* = 0.057) (Figure 5A). Spline-based Cox regression further demonstrated an approximately linear inverse association between QUICKI and cardiovascular mortality risk: lower QUICKI values were linked to elevated hazard ratios, while higher QUICKI values were progressively protective (Figure 5B). At higher values (≥0.40), the confidence intervals widened substantially, reflecting limited data density in the tails. Together, these findings confirm that QUICKI retained prognostic relevance in an independent, population-based dataset, reinforcing its value as a sensitive marker of cardiovascular risk beyond conventional metabolic indicators.

## Discussion

### Prognostic value of QUICKI across contexts

In this study of patients with ischemic cardiomyopathy undergoing revascularization, we demonstrated that insulin sensitivity, quantified by QUICKI, was consistently associated with postoperative ventricular remodeling and long-term cardiovascular mortality. While conventional metabolic markers such as fasting glucose, insulin, and lipid parameters showed limited discriminatory value, QUICKI emerged as a comparatively stronger predictor across analytic frameworks. It was stably selected in penalized regression, contributed significantly to linear effects in GLMNET, and showed favorable early time-dependent ROC performance. These findings were further reinforced in the external validation cohort derived from NHANES 2013–2016, where QUICKI also demonstrated prognostic relevance for cardiovascular mortality despite the population being generally healthy. This consistency across contexts suggests QUICKI's broader utility as a marker of metabolic vulnerability beyond overt disease.

### Complementary insights from analytic frameworks

The analytic frameworks provided complementary insights. GLMNET emphasized QUICKI's independent contribution to mortality risk, reflecting its role as a global metabolic signal across heterogeneous clinical backgrounds. In contrast, LightGBM prioritized nonlinear interactions involving renal function, myocardial injury, and markers of structural remodeling, placing QUICKI lower in importance. This divergence highlights a conceptual distinction: QUICKI may capture a pervasive metabolic phenotype that influences outcomes broadly, whereas nonlinear model emphasizes subgroup-specific pathways that may predominate in certain clinical contexts. The stability of QUICKI along the LASSO coefficient path further supports its stability as a prognostic marker.

### Extension beyond prior clinical research

Existing clinical research has predominantly applied QUICKI to characterize systemic insulin sensitivity in metabolic diseases, obesity, and endocrine dysfunction (6). QUICKI correlates with clamp-measured insulin sensitivity and has been used to stratify metabolic risk in obesity, prediabetes, and diabetes (21, 22). Cardiovascular studies have reported that insulin resistance is associated with coronary microvascular dysfunction, impaired diastolic relaxation and increased arterial stiffness, leading to adverse outcomes in patients with heart failure (23–27). However, these investigations focused on early metabolic abnormalities or non-ischemic populations. Our study suggests that QUICKI may have potential applicability in population with ischemic cardiomyopathy undergoing revascularization, indicating its associations with ventricular remodeling and long-term prognosis.

### Biological link between insulin sensitivity and remodeling

The inverse associations between QUICKI and changes in LVEDD and LVESD are consistent with a potential biological link between metabolic dysregulation and structural remodeling. Prior studies suggest that insulin resistance can initiate metabolic and cellular disturbances that may predispose the myocardium to maladaptive remodeling (25). Reduced insulin sensitivity has been reported to impair glucose uptake and shift substrate utilization toward fatty acid oxidation, an energetically inefficient process that can generate toxic lipid intermediates (28). These metabolic shifts have been associated with mitochondrial dysfunction and excess reactive oxygen species production (29, 30). Insulin resistance has also been linked to systemic inflammation, activation of the renin-angiotensin-aldosterone system, and increased sympathetic drive, which may collectively contribute to extracellular matrix turnover, collagen deposition, and chamber dilatation (24, 31–33). Lower insulin sensitivity has been associated with increased LV mass and reduced myocardial compliance (26, 34). Our findings are directionally consistent with this metabolic-structural continuum, suggesting that reduced insulin sensitivity may be associated with a greater tendency toward ventricular dilatation even after anatomically successful revascularization.

### Integration with the CKM framework

Within the contemporary Cardiovascular-Kidney-Metabolic (CKM) framework proposed by the American Heart Association (35), insulin resistance and related metabolic dysfunction sit within the Metabolic domain and interact with renal and cardiovascular processes; ventricular remodeling after ischemia is a downstream cardiovascular manifestation. By showing that lower QUICKI is associated with adverse remodeling after revascularization, our results provide evidence that this simple fasting-based index maps onto the metabolic-cardiovascular axis and position QUICKI as a candidate CKM metabolic marker for structural cardiac outcomes.

### Other metabolic indices

Other metabolic indices have also been investigated in cardiovascular settings. The triglyceride-glucose (TyG) index has been reported to associate with adverse outcomes across HF phenotypes (36, 37). Glycated hemoglobin (HbA1c) has been linked not only to cardiovascular risk in diabetic and non-diabetic populations, but also to mortality and hospitalization in HF (38). These markers largely reflect chronic glycemic exposure or lipid-glucose interactions and have primarily been linked to functional deterioration rather than structural cardiac changes. In our study, QUICKI showed early prognostic value and exhibited clear inverse associations with ventricular dimensions in patients with ischemic cardiomyopathy. These findings suggest that QUICKI may capture aspects of metabolic dysregulation that are more proximal to the metabolic-structural interface relevant to ischemic cardiomyopathy, complementing rather than duplicating the information provided by TyG or HbA1c.

### Study limitations and future directions

Several limitations must be acknowledged. This study was retrospective and conducted at a single center, which limits causal inference and generalizability. Although the sample size was fixed by available cases, the number of cardiovascular deaths (*n* = 128) provides adequate power to detect moderate effect sizes (detectable HR ≈ 1.45) at a two-sided *α* of 0.05. The sample size and number of cardiovascular deaths may have reduced the ability to detect complex nonlinear effects or higher-order interactions. The external validation using NHANES was performed in a generally healthy population with fewer events, potentially reducing statistical precision. In addition, the study did not comprehensively characterize renal burden, which constrains our ability to evaluate how kidney status may influence or modify the relationship between insulin sensitivity and ventricular remodeling within the CKM axis. Future research should include larger, multicenter cohorts with serial metabolic and imaging assessments to clarify dynamic trajectories and to determine whether improving insulin sensitivity can modify remodeling or long-term outcomes. To further situate these findings within the CKM framework, subsequent studies may also consider incorporating basic renal measures alongside metabolic and structural cardiac evaluation.

## Conclusions

QUICKI appears to be a practical, integrative marker of systemic insulin sensitivity with potential implications for ventricular remodeling and long-term prognosis. Within the contemporary CKM framework, our findings suggest that QUICKI may help operationalize the metabolic component linked to adverse cardiovascular remodeling. Rather than establishing clinical utility, these results support further evaluation of metabolic profiling in risk stratification for ischemic cardiomyopathy.

## Data Availability

The raw data supporting the conclusions of this article will be made available by the authors, without undue reservation.
