# Supplementary material for: Insulin sensitivity and cardiovascular risk after revascularization: associations with remodeling and long-term outcomes
Source: Front Cardiovasc Med. 2026 Jul 27;13:1796332. doi: 10.3389/fcvm.2026.1796332 (PMC13453814; doi:10.3389/fcvm.2026.1796332)
Supplement: Supplementary file 1 [file Table1.docx]

**Supplemental Table 1.** **Baseline characteristics by survival status.**

| **Characteristic** | **Alive N=604** | **Dead N=128** | **P value** |
| --- | --- | --- | --- |
| Gender |  |  | 0.621^a^ |
| Female | 77 (13%) | 19 (15%) |  |
| Male | 527 (87%) | 109 (85%) |  |
| Age (years) | 62 ± 10 | 69 ± 9 | <0.001^c^ |
| Drinking |  |  | 0.350^b^ |
| Current | 124 (21%) | 21 (16%) |  |
| Former | 18 (3.0%) | 6 (4.7%) |  |
| Never | 462 (76%) | 101 (79%) |  |
| Baseline LVEF (%) | 46 ± 7 | 42 ± 7 | <0.001^c^ |
| Follow-up LVEF (%) | 51 ± 9 | 46 ± 9 | <0.001^c^ |
| Baseline LVEDD (mm) | 58 ± 7 | 60 ± 7 | <0.001^c^ |
| Follow-up LVEDD (mm) | 56 ± 6 | 59 ± 8 | <0.001^c^ |
| Baseline LVESD (mm) | 44 ± 7 | 47 ± 7 | <0.001^c^ |
| Follow-up LVESD (mm) | 41 ± 7 | 45 ± 8 | <0.001^c^ |
| Baseline LAD (mm) | 42.3 ± 4.5 | 44.1 ± 5.5 | <0.001^c^ |
| Follow-up LAD (mm) | 41.5 ± 4.1 | 43.3 ± 5.4 | <0.001^c^ |
| BMI (kg/m²) | 24.9 ± 6.8 | 24.4 ± 3.4 | 0.267^c^ |
| Weight (kg) | 70 ± 11 | 69 ± 12 | 0.262^c^ |
| Height (m) | 1.68 ± 0.07 | 1.68 ± 0.07 | 0.384^c^ |
| Diabetes | 264 (44%) | 60 (47%) | 0.577^a^ |
| Hypertension | 421 (70%) | 96 (75%) | 0.276^a^ |
| Dyslipidemia | 288 (48%) | 58 (45%) | 0.696^a^ |
| Chronic kidney disease | 54 (8.9%) | 20 (16%) | 0.034^a^ |
| COPD | 55 (9.1%) | 20 (16%) | 0.040^a^ |
| Asthma | 7 (1.2%) | 5 (3.9%) | 0.042^b^ |
| Depression | 3 (0.5%) | 1 (0.8%) | 0.537^b^ |
| Cancer | 22 (3.6%) | 7 (5.5%) | 0.476^a^ |
| Thyroid dysfunction | 9 (1.5%) | 2 (1.6%) | 1.000^b^ |
| Peripheral arterial disease | 110 (18%) | 41 (32%) | <0.001^a^ |
| Comorbidity count |  |  |  |
| 0 | 50 (8.3%) | 11 (8.6%) |  |
| 1 | 149 (25%) | 21 (16%) |  |
| 2 | 204 (34%) | 30 (23%) |  |
| 3 | 147 (24%) | 41 (32%) |  |
| 4 | 38 (6.3%) | 21 (16%) |  |
| 5 | 13 (2.2%) | 2 (1.6%) |  |
| 6 | 3 (0.5%) | 2 (1.6%) |  |
| eGFR (mL/min/1.73m²) | 79 ± 20 | 69 ± 20 | <0.001^c^ |
| Gensini score | 90 ± 40 | 95 ± 44 | 0.275^c^ |
| Revascularization type |  |  | 0.196^a^ |
| CABG | 285 (48%) | 70 (55%) |  |
| PCI | 246 (42%) | 50 (39%) |  |
| Hybrid | 58 (9.8%) | 7 (5.5%) |  |
| Total cholesterol (mmol/L) | 4.08 ± 1.23 | 4.02 ± 0.98 | 0.547^c^ |
| HDL-C (mmol/L) | 0.98 ± 0.24 | 0.96 ± 0.24 | 0.598^c^ |
| LDL-C (mmol/L) | 2.46 ± 1.01 | 2.45 ± 0.81 | 0.848^c^ |
| Triglycerides (mmol/L) | 1.66 ± 0.98 | 1.57 ± 0.81 | 0.336^d^ |
| Neutrophils (%) | 65 ± 11 | 64 ± 11 | 0.488^c^ |
| Lymphocytes (%) | 24 ± 10 | 24 ± 10 | 0.882^c^ |
| WBC (10^9/L) | 7.72 ± 2.96 | 7.22 ± 2.20 | 0.030^c^ |
| Hemoglobin (g/L) | 132 ± 16 | 127 ± 16 | 0.004^c^ |
| Platelets (10^9/L) | 195 ± 65 | 183 ± 58 | 0.030^c^ |
| Fasting glucose (mmol/L) | 6.40 ± 2.29 | 6.25 ± 2.32 | 0.519^c^ |
| HbA1c (%) | 6.72 ± 1.48 | 6.95 ± 1.73 | 0.182^c^ |
| Fasting insulin (µU/mL) | 13 ± 16 | 14 ± 19 | 0.655^c^ |
| BUN (mmol/L) | 6.33 ± 2.57 | 7.17 ± 3.03 | 0.004^c^ |
| Creatinine (µmol/L) | 91 ± 43 | 101 ± 33 | 0.006^c^ |
| NT-proBNP (pg/mL) | 2,297 ± 2,831 | 2,871 ± 3,065 | 0.035^d^ |
| cTnI (ng/mL) | 16 ± 60 | 16 ± 112 | 0.967^c^ |
| Left main disease | 152 (25%) | 45 (35%) | 0.028^a^ |
| Family history | 95 (16%) | 17 (13%) | 0.573^a^ |
| ACE inhibitor | 218 (36.1%) | 50 (39.1%) | 0.526^a^ |
| ARB | 380 (62.9%) | 91 (71.1%) | 0.079^a^ |
| Beta-blocker | 560 (92.7%) | 119 (93%) | 0.920^a^ |
| Loop diuretics | 370 (61.3%) | 87 (68%) | 0.154^a^ |
| Thiazide diuretics | 96 (15.9%) | 15 (11.7%) | 0.232^a^ |
| Digoxin | 20 (3.3%) | 9 (7%) | 0.050^a^ |
| Aspirin | 543 (89.9%) | 116 (90.6%) | 0.804^a^ |
| Clopidogrel | 323 (53.5%) | 78 (60.9%) | 0.123^a^ |
| Nitrates | 438 (72.5%) | 95 (74.2%) | 0.694^a^ |
| Statins | 567 (93.9%) | 120 (93.8%) | 0.958^a^ |
| Calcium channel blocker | 55 (9.1%) | 16 (12.5%) | 0.239^a^ |

Baseline demographic, clinical, echocardiographic, metabolic, and laboratory characteristics are presented for patients alive (n = 604) and dead (n = 128) at follow‑up. Continuous variables are expressed as mean ± SD, and categorical variables as counts (percentages). P values were calculated using chi‑square tests (a), Fisher’s exact tests (b), Student’s t tests (c), or Wilcoxon rank‑sum tests (d), as appropriate.
