# Supplementary material for: Insulin sensitivity and cardiovascular risk after revascularization: associations with remodeling and long-term outcomes
Source: Front Cardiovasc Med. 2026 Jul 27;13:1796332. doi: 10.3389/fcvm.2026.1796332 (PMC13453814; doi:10.3389/fcvm.2026.1796332)
Supplement: Supplementary file 2 [file Table2.docx]

**Supplemental Table 2. Model performance on the test set.**

| Model | Test_AUC | Brier | Threshold | Sensitivity | Specificity | PPV | NPV |
| --- | --- | --- | --- | --- | --- | --- | --- |
| Blend | 0.790 | 0.1241 | 0.175 | 0.746 | 0.763 | 0.938 | 0.387 |
| LightGBM | 0.775 | 0.1250 | 0.165 | 0.834 | 0.658 | 0.921 | 0.455 |
| GLMNET | 0.767 | 0.1296 | 0.151 | 0.558 | 0.895 | 0.962 | 0.298 |
| XGB | 0.761 | 0.1254 | 0.161 | 0.657 | 0.789 | 0.937 | 0.326 |
| SVM | 0.698 | 0.1334 | 0.202 | 0.779 | 0.553 | 0.892 | 0.344 |

Discrimination, calibration, and classification metrics are reported for individual models and the weighted ensemble (Blend). Test AUC and Brier score reflect overall discrimination and calibration, respectively. Thresholds were selected based on optimal Youden index. Sensitivity, specificity, positive predictive value (PPV), and negative predictive value (NPV) are shown for each model.
