# Supplementary material for: Insulin sensitivity and cardiovascular risk after revascularization: associations with remodeling and long-term outcomes
Source: Front Cardiovasc Med. 2026 Jul 27;13:1796332. doi: 10.3389/fcvm.2026.1796332 (PMC13453814; doi:10.3389/fcvm.2026.1796332)
Supplement: Supplementary file 3 [file Table3.docx]

**Supplemental Table 3. Top 10 variables by selection frequency in LASSO (5‑fold cross‑validation).**

| **Variable** | **Freq λ.1se** | **Freq λ.min** | **Freq entry** |
| --- | --- | --- | --- |
| Age | 0.956 | 1.000 | 1.000 |
| Follow-up LVEF | 0.860 | 0.972 | 0.956 |
| Comorbidity count | 0.542 | 0.980 | 0.934 |
| Left main disease | 0.344 | 0.918 | 0.788 |
| Baseline LVEF | 0.434 | 0.780 | 0.662 |
| eGFR | 0.456 | 0.624 | 0.580 |
| **QUICKI** | **0.076** | **0.730** | **0.498** |
| Follow-up LVESD | 0.282 | 0.348 | 0.362 |
| Follow-up LAD | 0.150 | 0.484 | 0.318 |
| Baseline LAD | 0.222 | 0.370 | 0.340 |

Selection frequencies of candidate predictors are shown across penalization levels in GLMNET. Freq λ.1se and Freq λ.min indicate the proportion of times each variable was selected at the one‑standard‑error and minimum penalty thresholds, respectively.
