# Supplementary material for: Insulin sensitivity and cardiovascular risk after revascularization: associations with remodeling and long-term outcomes
Source: Front Cardiovasc Med. 2026 Jul 27;13:1796332. doi: 10.3389/fcvm.2026.1796332 (PMC13453814; doi:10.3389/fcvm.2026.1796332)
Supplement: Supplementary file 4 [file Table4.docx]

**Supplemental Table 4. Time‑dependent AUCs for metabolic indicators.**

| Variable | 4 Year | 5 Year | 6 Year | 7 Year | 8 Year | 9 Year | 10 Year |
| --- | --- | --- | --- | --- | --- | --- | --- |
| QUICKI | 0.721 | 0.571 | 0.546 | 0.551 | 0.523 | 0.524 | 0.493 |
| Fasting Glucose | 0.361 | 0.400 | 0.334 | 0.379 | 0.432 | 0.434 | 0.464 |
| Fasting Insulin | 0.311 | 0.461 | 0.506 | 0.491 | 0.506 | 0.509 | 0.530 |
| TG | 0.289 | 0.427 | 0.426 | 0.452 | 0.453 | 0.402 | 0.377 |
| TC | 0.422 | 0.512 | 0.480 | 0.461 | 0.424 | 0.439 | 0.387 |
| HDL-C | 0.575 | 0.516 | 0.510 | 0.494 | 0.480 | 0.514 | 0.511 |
| LDL-C | 0.419 | 0.503 | 0.474 | 0.469 | 0.430 | 0.449 | 0.403 |
| BMI | 0.427 | 0.522 | 0.499 | 0.477 | 0.461 | 0.456 | 0.457 |

AUC values for cardiovascular death prediction are shown for QUICKI and conventional metabolic indicators across 4–10 years of follow‑up.
