# Supplementary material for: Insulin sensitivity and cardiovascular risk after revascularization: associations with remodeling and long-term outcomes
Source: Front Cardiovasc Med. 2026 Jul 27;13:1796332. doi: 10.3389/fcvm.2026.1796332 (PMC13453814; doi:10.3389/fcvm.2026.1796332)
Supplement: Supplementary file 5 [file Table5.docx]

**Supplemental Table 5. Associations of metabolic indicators with cardiac remodeling parameters.**

| Outcome | Predictor | Estimate | Std.error | P.value |
| --- | --- | --- | --- | --- |
| ΔLVEDD | QUICKI | -12.303 | 4.747 | 0.010 |
| ΔLVEDD | Fasting glucose | 0.183 | 0.124 | 0.140 |
| ΔLVEDD | Fasting insulin | 0.024 | 0.015 | 0.118 |
| ΔLVEDD | TG | -0.134 | 0.307 | 0.663 |
| ΔLVEDD | TC | -0.151 | 0.232 | 0.517 |
| ΔLVEDD | HDL-C | -0.612 | 1.150 | 0.595 |
| ΔLVEDD | LDL-C | -0.226 | 0.285 | 0.428 |
| ΔLVEDD | BMI | -0.006 | 0.032 | 0.853 |
| ΔLVEF | QUICKI | 14.771 | 8.759 | 0.093 |
| ΔLVEF | Fasting glucose | 0.202 | 0.227 | 0.374 |
| ΔLVEF | Fasting insulin | -0.028 | 0.028 | 0.315 |
| ΔLVEF | TG | 0.741 | 0.562 | 0.188 |
| ΔLVEF | TC | -0.212 | 0.426 | 0.619 |
| ΔLVEF | HDL-C | 1.344 | 2.107 | 0.524 |
| ΔLVEF | LDL-C | -0.673 | 0.521 | 0.197 |
| ΔLVEF | BMI | -0.033 | 0.058 | 0.568 |
| ΔLVESD | QUICKI | -13.352 | 5.850 | 0.023 |
| ΔLVESD | Fasting glucose | 0.094 | 0.152 | 0.537 |
| ΔLVESD | Fasting insulin | 0.027 | 0.019 | 0.149 |
| ΔLVESD | TG | -0.426 | 0.377 | 0.260 |
| ΔLVESD | TC | -0.016 | 0.286 | 0.955 |
| ΔLVESD | HDL-C | -0.869 | 1.413 | 0.539 |
| ΔLVESD | LDL-C | 0.144 | 0.350 | 0.680 |
| ΔLVESD | BMI | -0.012 | 0.039 | 0.768 |

Linear regression estimates (β coefficients), standard errors, and p values are shown for metabolic predictors in relation to changes in left ventricular end‑diastolic diameter (ΔLVEDD), ejection fraction (ΔLVEF), and end‑systolic diameter (ΔLVESD).
